# Complex regional pain syndrome 1 – the Swiss cohort study

**DOI:** 10.1186/1471-2474-9-92

**Published:** 2008-06-23

**Authors:** Florian Brunner, Lucas M Bachmann, Ulrich Weber, Alfons GH Kessels, Roberto SGM Perez, Johan Marinus, Rudolf Kissling

**Affiliations:** 1 Department of Physical Medicine and Rheumatology, Balgrist University Hospital, Zurich, Switzerland; 2 Horten Centre for patient oriented research, University of Zurich, Zurich, Switzerland; 3 Clinical Epidemiology and Medical Technology Assessment, University Hospital Maastricht, Maastricht, The Netherlands; 4 Department of Anaesthesiology, VU University Medical Center, Amsterdam, The Netherlands; 5 Research Institute for Extramural Medicine (EMGO), VU University Medical Center, Amsterdam, The Netherlands; 6 Department of Neurology, Leiden University Medical Center, Leiden, The Netherlands

## Abstract

**Background:**

Little is known about the course of Complex Regional Pain Syndrome 1 and potential factors influencing the course of this disorder over time. The goal of this study is a) to set up a database with patients suffering from suspected CRPS 1 in an initial stadium, b) to perform investigations on epidemiology, diagnosis, prognosis, and socioeconomics within the database and c) to develop a prognostic risk assessment tool for patients with CRPS 1 taking into account symptomatology and specific therapies.

**Methods/design:**

Prospective cohort study. Patients suffering from a painful swelling of the hand or foot which appeared within 8 weeks after a trauma or a surgery and which cannot be explained by conditions that would otherwise account for the degree of pain and dysfunction will be included. In accordance with the recommendations of International Classification of Functioning, Disability and Health (ICF model), standardised and validated questionnaires will be used. Patients will be monitored over a period of 2 years at 6 scheduled visits (0 and 6 weeks, 3, 6, 12, and 24 months). Each visit involves a physical examination, registration of therapeutic interventions, and completion of the various study questionnaires. Outcomes involve changes in health status, quality of life and costs/utility.

**Discussion:**

This paper describes the rationale and design of patients with CRPS 1. Ideally, potential risk factors may be identified at an early stage in order to initiate an early and adequate treatment in patients with increased risk for delayed recovery.

**Trial registration:**

Not applicable

## Background

Complex Regional Pain Syndrome (CRPS) is a painful condition with clinical features that include pain, sensory-, sudomotor and vasomotor disturbances, trophic changes and impaired motor function [1]. Symptoms usually appear after an initiating noxious event such as trauma or surgery [2,3]. The course varies from mild and self-limiting to chronic disease with a high impact on daily functioning and quality of life [4]. CRPS is often associated with a significant morbidity and loss of quality of life [5]. Just recently, Duman et al. reported that in Turkey almost a third of patients with CRPS are not returning to work [6]. In a retrospective chart review of 143 patients with CRPS who had been assessed and/or treated at a university-based pain centre in the United States, over half of all patients were involved in worker compensation claims [7] and a fifth of all patients in law suits [7].

Information regarding relevant prognostic factors in CRPS is sparse or difficult to interpret, due to the specificity of circumstances in which the information was collected [8]. For the initiation of CRPS 1 certain psychological characteristics such as depression, recent life events or emotional instability are considered to be prognostic factors [9]. The nature and severity of the initiating event does not seem to have a prognostic value on the course of the disease [10]. In the literature, the following parameters have been identified as prognostic factors for delayed recovery: duration of the complaints (more than 90 days), cold CRPS 1, passive coping style and poor controllability of the complaints [11,12].

The goal of this study is a) to set up a database with patients suffering from suspected CRPS 1 in an initial stage, b) to perform investigations on epidemiology, diagnosis, prognosis, and socioeconomics within the database and c) to develop a prognostic risk assessment tool for patients with CRPS 1 taking into account symptomatology and specific therapies. Ideally, risk factors are recognized that are present early in the disease course so that patients with increased risk for delayed recovery can be identified early and treated adequately to minimize persistent pain and functional disability.

## Methods/Design

### Study design

The prospective cohort study follows an observational study design with a consecutive registration of patients with CRPS 1 for two years. Thereafter, we intend to extend the follow up of this cohort pending on further funding.

### Study population

We will include all consenting eligible patients suffering from a painful swelling of the hand or foot which appeared within 8 weeks after a trauma or a surgery and which cannot be explained by conditions that would otherwise account for the degree of pain and dysfunction.

### Inclusion criteria

1. Painful swelling of the hand or foot which appeared within 8 weeks after a trauma or a surgery and which cannot be explained by conditions that would otherwise account for the degree of pain and dysfunction.

2. One single extremity is affected.

3. Sings and symptoms are still present at first visit.

4. Patients able to attend follow-up visits at Balgrist University Hospital, Zurich, Switzerland.

5. Age limit 18 years or older.

6. Signed informed consent.

### Rationale for inclusion criteria

Our inclusion criteria are set very broadly. This allows us to distinguish between those subjects with CRPS like manifestations and others fulfilling diagnostic criteria of current classification systems. This approach has several advantages. First we are able to develop statistical tools to identify manifestational profiles that are unlikely to develop CRPS. Moreover we are able to study differences in the prognosis of patients with CRPS like symptoms from patients fulfilling the CRPS criteria. Finally, our over inclusive enrolment has the potential to inform new classification systems for CRPS with a focus on prognosis rather than diagnosis.

### Exclusion criteria

1. Other conditions which appeared before the initiating event or which can explain signs and symptoms better than CRPS 1.

2. Signs and symptoms on more than one extremity.

3. Gravidity, lactation.

### National and international collaboration

After an initial period, we plan collaboration with other Swiss national medical centres and societies from medical specialists, insurance companies and patient societies. Internationally, we collaborate with the Trauma Related Neuronal dysfunction (TREND) research consortium from the Netherlands [13].

### Procedure

We will recruit participants through direct referral from primary care physicians and medical specialists in physical medicine & rehabilitation, rheumatologists, neurologists, pain specialists and orthopaedic surgeons. FB, UW and RK perform the screening visit with possible participants. Subjects who meet the eligibility criteria will receive further information concerning the study. In particular, the aims and methods will be explained after which written consent to participate will be sought. This trial has been approved by the local Ethics Committee of Balgrist University Hospital (Spezialisierte Unterkomission fur Orthopadie der Kantonalen Ethikkommission, Zurich, Switzerland, reference number EK01/2008).

Assessments will be performed after 6 weeks, 3 months, 6 months, 12 months, 18 months and 24 months. The study follows a strictly observational design in which the therapeutic intervention will be registered and classified according to the list below (see table 1) and treatment effects will be recorded for several health related outcomes. The focus will be on observing the course of the disorder over time and to identify significant risk factors that may change the course in a positive or negative way. Patients undergo individual treatment protocols based on best current internationally accepted guidelines [14-16]. In order to exclude other underlying causes that may account for the degree of pain or limitation exhibited by the patient, additional laboratory tests or imaging methods will be performed at the discretion of the physician. However, a minimal set of diagnostic tests will be obtained in every participants (C-reactive protein and conventional x-ray of both hands respectively feet). To determine the positive diagnosis of CRPS 1 we will use the IASP criteria [17]. Simultaneously, we will collect a broad set of CRPS associated signs and symptoms. This additional information will help us to classify the patients according to other diagnostic criteria sets (e.g. [10,18,19]) and allows us to perform future adaptations due to changing concepts in CRPS taxonomy.

**Table 1:** Categories of treatment

Pharmacological treatment:
Topical medications: Topical anaesthetics, topical capsaicin
Oral medications: Acetaminophen, NSAR, opioids, antidepressants, anticonvulsive medications
Other medications: Corticosteroids, calcitonin, bisphosphonates, vitamin c, nifedipin
Interventional therapies:
Sympathetic blockades, intravenous regional anaesthetic blocks, intravenous blocks, other blocks, neurolytic sympathetic blocks

Implanted therapies:
Peripheral nerve stimulation, spinal cord stimulator

Functional restoration:
Physical therapy, occupational therapy

Behavioural therapy:
Cognitive therapy, behavioural therapy

### Safety monitoring and adverse events

Since this study follows an observational design without an actual intervention there is no substantial risk associated with participation. There is no additional physical and physiological discomfort to be expected with participation. However, any unexpected events and adverse events will be registered and followed. The investigator will take care that all subjects are kept informed. All adverse events will be followed until they have abated, or until a stable situation has been reached. Depending on the event, follow up may require additional tests or medical procedures as indicated, and/or referral to the primary care physician or a medical specialist.

### Outcome measures (baseline & follow up)

**Figure 1:**
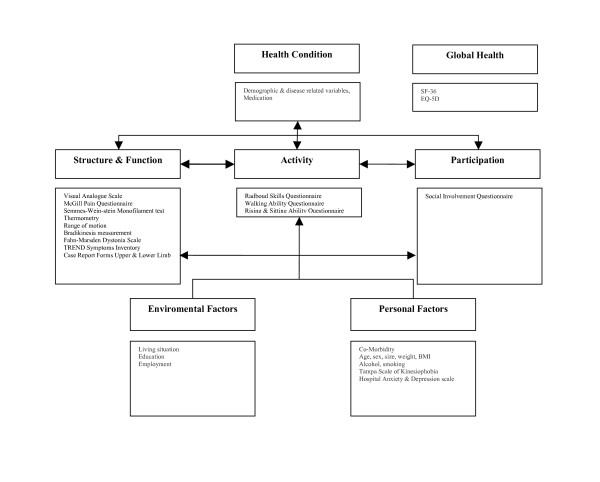
**Integrated research model based on ICF model (adapted from**[8]**).**

Figure 1 outlines the way how we will evaluate the health status of the participants. The assessments were chosen in order to cover the domains as described in the ICF model [20]. The consequences of CRPS 1 will be monitored at different levels such as body structure and function, activity and participation as well as contextual factors influencing a person's health condition. Table 2 summarizes the instrument used per evaluation point.

**Table 2:** Outcome measures (adapted from [8])

**Assessments and Instruments**	**Baseline**	**6 weeks**	**3 months**	**6 months**	**12 months**	**24 months**
**Baseline Variables**						
Demographic variables	x	x	x	x	x	x
Disease related variables	x					
Medication	x	x	x	x	x	x
Intoxication (smoking, alcohol)	x					
**Body Structure and Functions**						
*Pain*						
VAS diary pain	x	x	x	x	x	x
Mc Gill pain questionnaire	x	x	x	x	x	x
*Sensory symptoms*						
Sensory testing [22]	x	x	x	x	x	x
*Vasomotor*						
Thermometry [23]	x	x	x	x	x	x
*Sudomotor/edema*						
Indirect volumetry [24]	x	x	x	x	x	x
*Motor/trophic*						
Range of motion [25]	x	x	x	x	x	x
Dystonia (Fahn Marsden Scale) [26]^+^	x	x	x	x	x	x
TREND Symptoms Inventory [27]	x	x	x	x	x	x
**Participation**						
Radboud Skills Questionnaire [28]* °	x	x	x	x	x	x
Walking Ability Questionnaire [29]"°	x	x	x	x	x	x
Rising & Sitting Ability Questionnaire [30]"°	x	x	x	x	x	x
**Participation**						
Social Involvement Questionnaire [31]	x			x	x	x
**Global Outcomes**						
SF-36 [32]	x			x	x	x
EQ-5D [33]	x			x	x	x
**Environmental factors**						
Living situation, education, employment	x	x	x	x	x	x
**Personal Factors**						
Tampa Scale of Kinesiophobia [34]	x			x	x	x
Hospital Anxiety & Depression scale [35]	x			x	x	x
Co-Morbidity [36]	x			x	x	x

^+^The Fahn Marsden Score will only be used in patients with clinical signs of dystonia.

*Only in patients with CRPS 1 in the upper extremity.

"Only in patients with CRPS 1 in the lower extremity.

°These questionnaires will be validated in German as a side project.

### Socioeconomic Analysis

#### Cost analysis

Both direct health care costs, direct non-healthcare costs (patient cost) and indirect cost (productivity losses) will be included in the analysis. Direct health care costs include the costs of the diagnostic and prognostic trajectory and staff time (primary care physicians, specialists in physical medicine & rehabilitation, rheumatology, neurology, pain specialists and orthopaedic surgeons.). Volumes will be collected using existing registration systems and cost diaries. Non-medical costs such as productivity losses, time and travel costs and other out-of-pocket costs for undergoing diagnostic procedures or treatment will be registered using cost diaries. This diary will be handed over to patients upon their first visit. Cost calculation will be based on real prices or on unit prices from the Swiss Guideline for Cost Calculation. In case of household activities, or other unpaid activities foregone, shadow prices will be used.

### Sample size

Since this is a consecutive registration of eligible patients, a sample size calculation is not necessary. After enrolling the first 100 patients, we perform an internal quality assessment and, if necessary, perform procedural changes. When the value of a combination of several variables needs to be quantified no straight forward methods to estimate the required sample size are available. A frequently used "rule of thumb" recommends that for each variable included in the analysis, at least 10 patients with a positive outcome (in this case the resolution of symptoms or other outcomes) are necessary [21]. Based on the assumption that about one third of patients will not return to work (indicating chronification) and the assessment of about 30 covariates we will need to enrol about 700 patients.

### Statistical analysis

Epidemiologic data and patient characteristics available on continuous scales will be presented with medians and interquartile ranges or means and standard deviations as appropriate. Categorical data will be presented as rates and percentages.

Associations between individual (independent) variables and the outcome variables will be reported using correlation coefficients. Results from univariate analysis will inform multivariate modeling.

Assessment of causal associations will be performed using multivariate models including potential confounders along with the independent variables of interest. Prognostic scores will be built using either multivariate logistic regression analysis or Cox proportional hazard models. Models will be validated in cross samples. Calibration and discrimination of the Cross validated prognostic instruments will be assessed using the Brier Score. Missing data will be imputed using multiple imputation methods if the assumption that missingness is at random seems justifiable.

## Discussion

This paper describes the rationale and design of a prospective cohort study following the course of CRPS 1. We intend to set-up a prospectively acquired clinical database for two years. To our knowledge this is the first attempt to set up a prospective cohort study with CRPS 1 patients.

We believe that the project is timely. From a socioeconomic and epidemiological perspective many questions regarding CRPS remain unanswered. While considerable efforts have gone into basic science research dealing with pathogenesis of CRPS the clinical counterpart has not been vigorously fostered. Among the few examples we are aware of the Dutch consortium Trend which established a registry of cases with CRPS I. We think that our collaboration with this group has a significant synergistic capacity. Our partnership will contribute to a better understanding of many important aspects of CRPS and inform the future research agenda in this area. It is likely to generate considerable transfer value to the health care system not only because its results will have a direct impact on patient care but also because it allows identifying patients requiring particular attention and care. Finally, we believe that this study will guide investigators to set the research agenda in this rapidly evolving field.

## Abbreviations

CRPS 1: Complex regional pain syndrome 1; ICF: International Classification of Functioning; BMI: Body mass index; EQ-5D: EuroQol-5D; TREND: Trauma related neuronal dysfunction.

## Competing interests

The authors declare that they have no competing interests.

## Authors' contributions

All authors participated in trial design; FB and LMB drafted the protocol. UW, AGHK, RSGMP, JM and RK critically reviewed the protocol; UW, RK and FB obtained funding.

## Pre-publication history

The pre-publication history for this paper can be accessed here:


